# Combined Effects of Flood Disturbances and Nutrient Enrichment Prompt Aquatic Vegetation Expansion: Sediment Evidence from a Floodplain Lake

**DOI:** 10.3390/plants14152381

**Published:** 2025-08-02

**Authors:** Zhuoxuan Gu, Yan Li, Jingxiang Li, Zixin Liu, Yingying Chen, Yajing Wang, Erik Jeppesen, Xuhui Dong

**Affiliations:** 1 School of Geography and Remote Sensing, Guangzhou University, Guangzhou 510006, China; 18024943041@163.com (Z.G.); 17373614680@163.com (J.L.); lucasliu11@outlook.com (Z.L.); 32365400036@e.gzhu.edu.cn (Y.C.); 2 Centre for Climate and Environmental Changes, Guangzhou University, Guangzhou 510006, China; xhdong@gzhu.edu.cn; 3 Department of Ecoscience, Aarhus University, 8000 Aarhus, Denmark; ej@ecos.au.dk; 4 Sino-Danish Centre for Education and Research (SDC), Beijing 100049, China; 5 Institute for Ecological Research and Pollution Control of Plateau Lakes, School of Ecology and Environmental Science, Yunnan University, Kunming 650500, China

**Keywords:** lake sediment, aquatic macrophyte, plant macrofossil, nutrient enrichment, extreme flood

## Abstract

Aquatic macrophytes are a vital component of lake ecosystems, profoundly influencing ecosystem structure and function. Under future scenarios of more frequent extreme floods and intensified lake eutrophication, aquatic macrophytes will face increasing challenges. Therefore, understanding aquatic macrophyte responses to flood disturbances and nutrient enrichment is crucial for predicting future vegetation dynamics in lake ecosystems. This study focuses on Huangmaotan Lake, a Yangtze River floodplain lake, where we reconstructed 200-year successional trajectories of macrophyte communities and their driving mechanisms. With a multiproxy approach we analyzed a well-dated sediment core incorporating plant macrofossils, grain size, nutrient elements, heavy metals, and historical flood records from the watershed. The results demonstrate a significant shift in the macrophyte community, from species that existed before 1914 to species that existed by 2020. Unlike the widespread macrophyte degradation seen in most regional lakes, this lake has maintained clear-water plant dominance and experienced continuous vegetation expansion over the past 50 years. We attribute this to the interrelated effects of floods and the enrichment of ecosystems with nutrients. Specifically, our findings suggest that nutrient enrichment can mitigate the stress effects of floods on aquatic macrophytes, while flood disturbances help reduce excess nutrient concentrations in the water column. These findings offer applicable insights for aquatic vegetation restoration in the Yangtze River floodplain and other comparable lake systems worldwide.

## 1. Introduction

Floodplain lake ecosystems are recognized as being among the most valuable and unique ecosystems globally [1]. Aquatic macrophytes are a critical component of floodplain lake ecosystems and play a vital role in regulating aquatic environments and enhancing ecological services [2]. Studies have shown that submerged macrophytes not only improve water clarity by reducing sediment resuspension but also suppress phytoplankton growth through light shading, nutrient competition, and allelopathic effects, thereby maintaining clear water conditions [3,4,5]. Furthermore, they provide food and habitats for aquatic organisms, significantly contributing to prolonged food chains and enhanced biodiversity [6,7]. Consequently, restoring submerged macrophyte communities to restructure ecosystem frameworks is regarded as one of the most effective restoration strategies for shallow lakes [8].

Floodplain lakes exhibit pronounced flood pulse effects, where periodic flooding events drive shifts in aquatic macrophyte vegetation composition and biomass dynamics [9]. Floods significantly reduce water transparency through combined effects of water level elevation, external sediment inputs, and sediment resuspension, suppressing photosynthetic activity in macrophytes [10,11]. Simultaneously, external nutrient inputs (e.g., nitrogen and phosphorus) from catchments transported by floods alter lake nutrient stoichiometry, potentially exacerbating eutrophication [12,13]. Furthermore, hypoxic conditions induced by flooding interfere with oxygen metabolism in plant tissues, while strong hydrodynamic forces damage the structural integrity and nutrient uptake capacity of the aquatic macrophyte root systems [14,15].

Growth and reproduction of aquatic macrophytes are typically regulated by nutrient dynamics. Essential nutrients such as nitrogen and phosphorus, when moderately enriched [16], can enhance macrophyte proliferation. However, excessive enrichment of exogenous nutrients often triggers lake eutrophication, leading to a substantial increase in phytoplankton biomass. Such algal blooms reduce water transparency through light shading, suppressing photosynthetic activity in macrophytes. Additionally, secondary metabolites released during algal metabolism may exert toxic effects on aquatic macrophytes [17].

Hydrological alterations and elevated nutrient loading have been identified as key drivers of the global decline in aquatic macrophyte distribution and species diversity. Over the past half-century, approximately 65.2% of lakes worldwide have experienced reductions in macrophyte coverage [18], with 12.8% experiencing extensive degradation or even complete loss of aquatic vegetation [19]. According to the Intergovernmental Panel on Climate Change (IPCC) Sixth Assessment Report, the frequency and intensity of extreme flood events are projected to increase significantly [20]. Concurrently, global lake eutrophication continues to intensify, particularly in developing regions of Asia and Africa [21,22]. Under these dual pressures, macrophyte communities face heightened survival challenges. Consequently, the mechanisms by which flood disturbances and nutrient enrichment impact macrophyte communities have become a central research focus in aquatic plant ecology.

However, due to the lack of long-term monitoring (generally <50 years) of aquatic macrophyte evolution in most Chinese lakes, the current mechanistic understanding of their responses to flood disturbances and nutrient enrichment primarily relies on historical surveys and experimental simulations, lacking empirical validation from long-term historical records [23,24]. This gap impedes a deeper exploration of the flood and nutrient impacts on macrophytes. Recent advances in paleolimnology show that analyzing plant macrofossils preserved in lake sediments can reconstruct the long-term succession of historical aquatic plant communities [25]. Unlike pollen, well-preserved plant macrofossils (including seeds, fruits, leaves, bud scales, and cuticles) offer superior taxonomic precision and local distribution patterns, enabling more accurate species-level identification. This enhanced resolution allows for refined classification of plant community types, making macrofossils particularly valuable for both paleoecological reconstructions and informing aquatic ecosystem restoration efforts [26].

To elucidate the impacts of flood disturbances and nutrient enrichment on aquatic macrophytes, this study selected Huangmaotan Lake, a shallow floodplain lake in the Yangtze River Basin, as the research site. By employing high-resolution dating techniques and analyzing plant macrofossils, we reconstructed historical aquatic vegetation dynamics in the lake. Combined with sedimentary indicators such as grain size and elemental geochemistry, this study aimed to unravel the effects of historical flood disturbances and nutrient enrichment on macrophyte communities, providing a theoretical foundation for aquatic vegetation restoration in floodplain lakes across the middle and lower Yangtze River. Our hypotheses were as follows: (1) Over the past two centuries, shifts in hydrological conditions and nutrient status have jointly driven the succession of macrophyte communities in Huangmaotan Lake, leading to a transition from species adapted to stable oligotrophic environments to those favoring disturbed oligo-mesotrophic conditions. (2) Combined effects of flood disturbances and nutrient enrichment critically influence macrophyte dynamics, potentially underpinning the lake’s sustained clear water state.

## 2. Materials and Methods

### 2.1. Study Site

**Figure 1:**
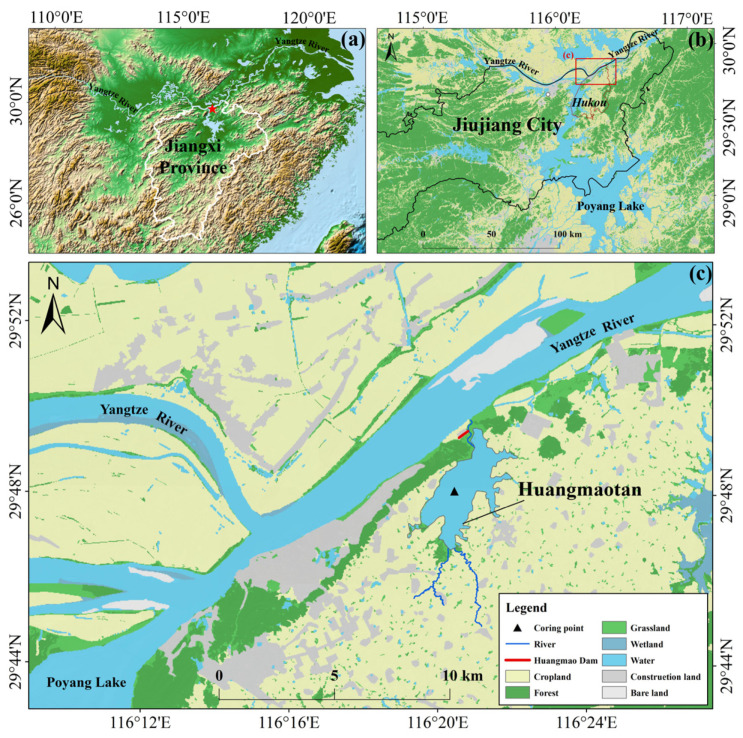
Location and sampling sites in Huangmaotan Lake. (**a**) Topography of Jiangxi Province, China; (**b**) location of Hukou County in Jiujiang City and its surrounding hydrological environment; (**c**) land use around Huangmaotan Lake.

Huangmaotan Lake (29°46′–29°49′ N, 116°19′–116°22′ E) is a freshwater lake situated on the southern bank of the middle and lower reaches of the Yangtze River in Hukou County, Jiujiang City, Jiangxi Province. The lake covers a water surface area of approximately 7.72 km^2^ (Figure 1). The lake exhibits pronounced seasonal water level fluctuations. It has maximum water depths of 3–4 m. The lake is located in the south subtropical maritime monsoon climatic zone, with an average temperature of 17.4 °C and an average annual rainfall of 1398.7 mm. The concentrated precipitation within the basin frequently triggers flood events during summer [27]. The lake receives freshwater from two rivers, the Siguan River and the Shijia River, and connects to the Yangtze River through a northern river that naturally drains the lake.

### 2.2. Historical Records on Droughts and Floods

We compiled historical flood records from the Huangmaotan watershed. Given the watershed’s location within Hukou County, Jiujiang City, the precipitation data were primarily sourced from the Jiujiang City Meteorological Station and the hydrological records of Hukou County. First, the historical drought–flood grades of the Huangmaotan Lake watershed were extracted from the Atlas of Drought and Flood Distribution in China in the Recent 500 Years [28,29]. Subsequently, based on flood records from the Hukou County Annals and China Meteorological Disaster Compendium: Jiangxi Volume, omissions of major flood events in the existing atlas were supplemented and corrected, resulting in a reconstructed drought–flood grade series for the period 1800–2000 [27,30]. To ensure the consistency of the drought–flood sequence, the drought–flood grade was calculated with cumulative precipitation from May to September 2000–2020 at Jiujiang City. The drought–flood grades were calculated using the standardized methodology from the Atlas of Drought and Flood Distribution in China in the Recent 500 Years, with the following formula:

(1) $$\mathrm{Flood}:\;R_{i}\;>\;(\bar{R}\;+\;1.17\sigma )$$

(2) $$\mathrm{Partial}\;\mathrm{flood}:\;(\bar{R}+0.33\sigma )\;<\;R_{i}\;\leq \;(\bar{R}\;+\;1.17\sigma )$$

(3) $$\mathrm{Normal}:\;(\bar{R}-0.33\sigma )\;<\;R_{i}\;\leq \;(\bar{R}\;+\;0.33\sigma )$$

(4) $$\mathrm{Partial}\;\mathrm{drought}:\;(\bar{R}-1.17\sigma )\;<\;R_{i}\;\leq \;(\bar{R}\;-\;0.33\sigma )$$

(5) $$\mathrm{Drought}:\;R_{i}\;\leq \;(\bar{R}\;-\;1.17\sigma )$$

*R_i_* was the cumulative precipitation from May to September at Huangmaotan Lake watershed year by year; $\bar{R}$ was multi-year mean precipitation for May to September (2000–2020); σ was the standard deviation of May to September precipitation (2000–2020).

Precipitation data for the Huangmaotan Lake watershed (2000–2020) were obtained from the Environmental Meteorology Data Service Platform (http://eia-data.com/, accessed on 2 May 2025). The drought–flood grade series of Huangmaotan Lake from 1800 to 2020 was ultimately established. To better characterize flood variability, the grades were classified as follows: Drought-grade 1; Partial drought-grade 2; Normal-grade 3; Partial flood-grade 4; Flood-grade 5. To filter out high-frequency fluctuations, the original drought–flood data were processed using a 5-year moving average (MA5). Furthermore, for clearer identification of flood-prone periods, a 10-year sliding window analysis was implemented. The flood frequency threshold was determined by calculating the sum of the mean and standard deviation across all windows. Periods exceeding this threshold were classified as high-frequency flood periods. This method systematically identified continuous flood-prone periods in the Huangmaotan Lake watershed between 1800 and 2020.

### 2.3. Historical Lake Area and Shoreline Changes Extracted from Remote Sensing Images

Historical lake areas and shorelines were obtained from Landsat imagery provided by the United States Geological Survey (USGS: https://www.usgs.gov/, accessed on 24 March 2025) [31]. A total of five periods of remote sensing imagery were collected, including Landsat-1 MSS images (1973), Landsat-5 MSS images (1985), Landsat-5 TM images (2000), and Landsat-8 OLI images (2010 and 2020). Only the remote sensing images acquired during non-flood seasons (January–April and October–December) in the Yangtze River basin were selected to ensure scientific comparability of lake area measurements. The remote sensing images underwent radiometric correction and geometric rectification before being assigned a defined projection. Lake areas and shoreline were then calculated using the Geometry Calculator tool in ArcGIS.

### 2.4. Sampling and Laboratory Analysis

A sediment core (HTM2, location 29°47′58″ N, 116°20′32″ E) was collected using a UWITEC gravity corer in the Huangmaotan Lake center in July 2019. Field samples were subsampled at 2 cm intervals onsite, sealed in polyethylene bags, and transported to the laboratory for refrigeration at 4 °C. Samples from the top 42 cm of the sediment core were analyzed, including measurements of radionuclide activity (^210^Pb and ^137^Cs), particle size, heavy metal elements (e.g., Cu, Zn, Pb, Ti, Fe), total phosphorus (TP), and plant macrofossils. 

^210^Pb and ^137^Cs activities were measured by direct γ spectrometry using an Ortec HPGe GWL series of well-type, coaxial, low background, and intrinsic germanium detectors (HPGe GWL-120-15, EG&G, Oak Ridge, TN, USA). Following the composite Constant Rate of Supply (CRS) model [32], we segmented the core into upper and lower sections using the 1963 ^137^Cs activity peak as a chronological marker. By integrating ^210^Pbex activity profiles with section-specific formulae, we reconstructed the chronological framework for Huangmaotan Lake [33].

Particle size measurements were conducted using a Malvern Mastersizer 2000 laser particle-size analyzer (Mastersizer-2000; Malvern, UK). Following pretreatment with 10% HCl for carbonate removal and 30% H_2_O_2_ with sodium hexametaphosphate as a dispersing agent for organic matter elimination, the measurements yielded particle size distributions. After pretreatment, particle size distributions were measured and categorized into clay (<4 μm) and silt (4–64 μm) fractions [34]. The median particle size (MD) was then calculated to represent the central tendency of the distribution. The sand fraction (>64 μm) constituted less than 1% and was therefore considered negligible in the subsequent analyses.

For geochemical analysis, samples (2 cm intervals) were air-dried at ambient temperature (<50 °C), homogenized through a 100 µm sieve, and stored in contamination-free containers. Subsequently, a suite of elements, including heavy metals (e.g., Cu, Zn, Pb, Ti, Fe) and TP were quantified via ICP-MS with matrix-matched calibration standards.

Plant macrofossil extraction followed standardized procedures [35]. For each sample, a measured sediment volume (20–50 cm^3^) was treated with dilute KOH solution to remove humic acids before sequential sieving through 250 μm and 125 μm mesh sieves. The entire 250 μm fraction was examined under a stereomicroscope (10–100× magnification), while a representative quarter aliquot of the 125–250 μm fraction was analyzed and scaled to total volume. Macrofossil concentrations were expressed as counts per 100 cm^3^ sediment (no./100 cm^3^). Identification of plant macrofossils primarily included seed, leaf, and fruit remains, with most specimens classifiable to species level. During quantification, complete macrofossils were counted as discrete units, while fragmentary specimens were proportionally enumerated based on preservation state (e.g., 0.5 units for 50% intact specimens). Taxonomic determinations referenced published morphological atlases by Berggren, Haas, Mauquoy & Van Geel [36,37,38] in conjunction with the Chinese Plant Species Information Database (http://db.kib.ac.cn/). Stratigraphic zonation and absolute concentration mapping were conducted using Tilia v3.0.3-CONISS and Tilia v3.0.3-Graph software packages.

### 2.5. Numerical Analyses

Information on plant community dynamics was obtained using Detrended Correspondence Analysis (DCA). The results, which involved the first axis length of 2.82 standard units, signaled a heightened degree of homogeneity in the alterations in species distribution. This finding aligns with the linear assumption underlying Principal Component Analysis (PCA). Consequently, using the first axis score of PCA was appropriate to represent the predominant trend in ecosystem state alteration. PCA analysis was employed to determine the change characteristics of sediment heavy metal elements. DCA and PCA were performed in R 4.1.0 using the vegan package.

Pearson correlation analysis was performed on plant macrofossil PC1 (PC1−P), heavy metal element PC1 (PC1−M), drought and flood grades, heavy metal elements, nutrient elements, particle size, and sedimentation rates. This analysis was conducted using SPSS 27.0.1, using *p*-values utilized to determine the statistical significance of the relationships. Subsequently, correlation matrices were plotted using Origin 2025.

## 3. Results

### 3.1. Historical Drought–Flood Variability in the Huangmaotan Watershed

**Figure 2:**
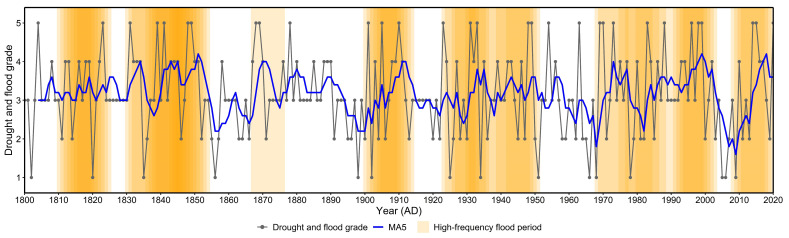
Drought–flood grade variations in Huangmaotan Lake watershed from 1800 to 2020. The color intensity of high-frequency flood periods corresponds to the number of overlapping sliding windows. MA5: 5-year moving average of drought–flood grades.

The Huangmaotan Lake watershed exhibited significant interannual variability in drought–flood grades from 1800 to 2020, with a gradually increasing trend (Figure 2). The interannual frequency of flood events in the 19th century was notably lower than in the post-19th-century period, and extreme flood events became more frequent after the 19th century. Calculations based on a 10-year sliding window revealed an average of 3.6 flood events per window during 1800–2020, with a standard deviation of 1.38. Consequently, windows exceeding 4.98 flood events (mean + 1 standard deviation) were classified as high-frequency flood periods. Analysis indicated high-frequency flood periods in the Huangmaotan Lake watershed from 1800 to 2020, specifically 1810s–1820s, 1830s–1850s, 1870s, 1900s–1910s, 1920s–1950s, 1970s–2000s, and 2010s–2020s. Notably, the 1920s–1950s and 1970s–2000s were periods of relatively continuous flood occurrences.

### 3.2. Historical Changes in Lake Morphology

**Figure 3:**
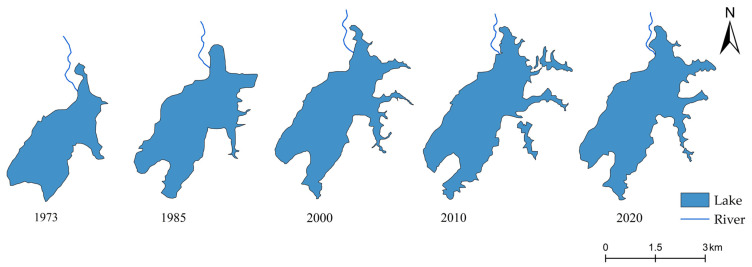
Morphological changes in Huangmaotan Lake in different periods.

Since 1973, Huangmaotan Lake has exhibited a characteristic pattern of rapid initial expansion followed by a slight decrease (Figure 3). Compared to 1973, the lake area significantly expanded by 1985, continued to grow until 2010, after which a slight reduction occurred. Similarly, the shoreline grew steadily until 2010, after which it began to contract (Table 1). The construction of the Huangmao Dam in the northern section has mitigated area and shoreline changes in that region, confining the major morphological transformations primarily to the southeastern sector.

### 3.3. Sediment Chronology

**Figure 4:**
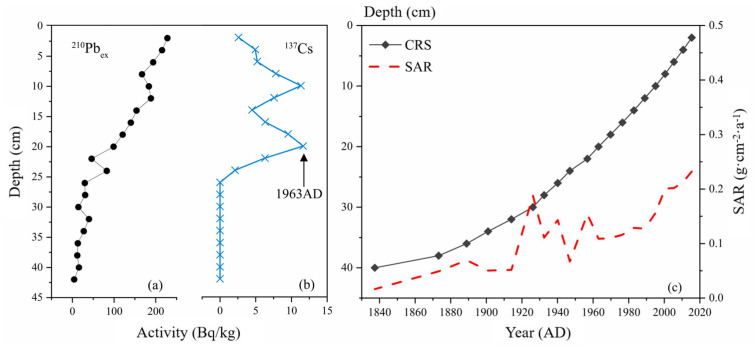
Dating results of the Huangmaotan Lake core (**a**) variation in 210Pb specific activity; (**b**) variation in 137Cs specific activity; (**c**) age-depth model and sedimentation rate.

A distinct ^137^Cs accumulation peak was observed at 20 cm depth in the Huangmaotan sediment core, marking the 1963–1964 depositional layer corresponding to the global nuclear testing maximum in 1963. At 26 cm depth, the onset of ^137^Cs accumulation was detected. Due to anthropogenic impacts on Huangmaotan Lake, sedimentation rates exhibited substantial variability, particularly during 1920–1960. Consequently, sediment chronology was calculated using the Constant Rate of Supply (CRS) model. The variation characteristics of ^210^Pb_ex_ and ^137^Cs activities with depth in the sediment core are presented in Figure 4a and Figure 4b, respectively. The age–depth relationship for the historical sediment core and sediment accumulation rate (SAR) are illustrated in Figure 4c.

### 3.4. Aquatic Plant Vegetation Assemblage in Huangmaotan Lake

**Figure 5:**
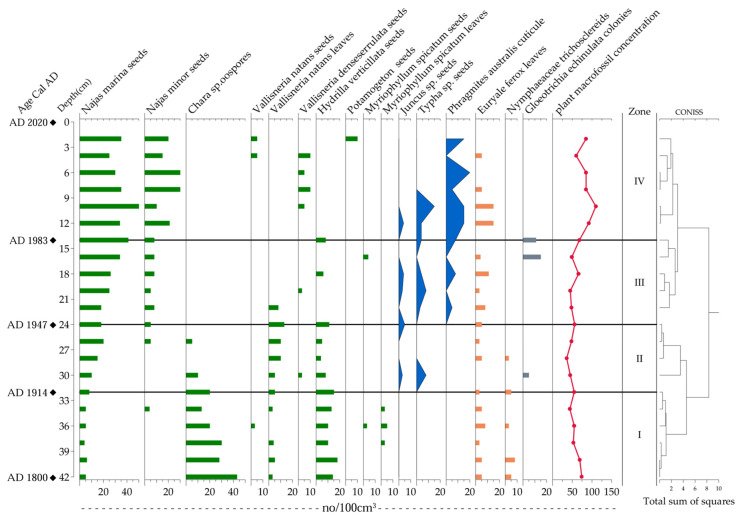
Stratigraphic plots of aquatic plant macrofossils in the Huangmaotan Lake core (green—submerged, blue—emergent, orange—floating-leaved, gray—cyanobacterial colonies).

Thirteen aquatic plant taxa were identified in the sediment core HTM2, representing three life forms, including emergent macrophytes, submerged macrophytes, and floating-leaved plants, with submerged species dominating the assemblage (Figure 5). Emergent macrophytes included *Juncus* sp., *Typha* sp. and *Phragmites australis*. Submerged macrophytes comprised *Najas marina*, *Najas minor*, *Hydrilla verticillata*, *Vallisneria natans*, *Vallisneria denseserrulata*, *Myriophyllum spicatum*, *Chara* sp. and *Potamogeton* sp. Floating-leaved plants consisted of *Euryale ferox* and *Nymphaeaceae*. Additionally, the cyanobacterium *Gloeotrichia echinulata* was present in the sediment record.

Using the CONISS method, the sediment core can be divided into four assemblage zones (from bottom to top) based on changes in plant macrofossil abundance, described as follows:

Zone I (42–32 cm; ca. 1800–1914 AD): In this period, abundant remains of *Chara* sp. and *H. verticillata* were identified. Remains of *N. marina* and *V. natans* were consistently present, but in low abundances, with occasional occurrences of *M. spicatum* remains. Floating-leaved plant remains, such as *Euryale ferox*, were regularly recorded, while *Nymphaeaceae* remains appeared sporadically. Notably, no remains of emergent plants were detected. In general, the aquatic vegetation was dominated by *Chara* sp. and *H. verticillata*, with co-occurring submerged macrophytes, including *N. marina* and *V. natans*, as well as the floating-leaved species *Euryale ferox*.

Zone II (32–24 cm; ca. 1914–1947 AD): This period was characterized by a marked decline or disappearance of *Chara* sp. remains, contrasting with increased abundances of *N. marina* and *V. natans*. Emergent plant remains (*Juncus* sp. and *Typha* sp.) were first recorded in this zone, albeit in low concentrations, while floating-leaved macrophytes exhibited stable remnant abundances. In general, the aquatic vegetation was dominated by *N. marina*, *H. verticillata*, and *V. natans*, with relatively stable populations of floating-leaved plants. Noteworthy is the initial appearance of small amounts of emergent vegetation in the lake during this period.

Zone III (24–14 cm; ca. 1947–1982 AD): This period witnessed a substantial increase in *N. marina* and *N. minor* remains compared to the preceding zone, accompanied by a significant decrease in *H. verticillata* remains. Notably, *Chara* sp. and *V. natans* remains disappeared completely. Emergent plant remains showed marked enrichment, with abundant *P. australis* appearing for the first time. While *Nymphaeaceae* remains vanished, *Euryale ferox* persisted with a consistently high abundance. Additionally, elevated concentrations of *Gloeotrichia echinulata* remains were observed. In general, this period featured high macrophyte diversity with pronounced expansion of emergent vegetation. The dominant community structure shifted notably, establishing an *N. marina* and *N. minor* assemblage as the new predominant aquatic vegetation.

Zone IV (14–0 cm; ca. 1982–2020 AD): Compared to the previous zone, *N. marina* and *N. minor* remains showed continued numerical increases, while *V. denseserrulata* remains exhibited a substantial increase. The sediment surface layer displayed the first appearance of *Potamogeton* sp. remains and the return of *V. natans* remains. For emergent plants, *P. australis* remains demonstrated a steady rise in abundance. Remains of the floating-leaved species *Euryale ferox* maintained a consistent presence but showed a diminishing trend. To summarize this period, emergent macrophytes increased in abundance while *Euryale ferox* declined gradually, forming an aquatic community dominated by *Najas marina*, *N. minor*, and *P. australis*, with *V. denseserrulata* as a subdominant component.

### 3.5. Multi-Indicator Analysis Results

**Figure 6:**
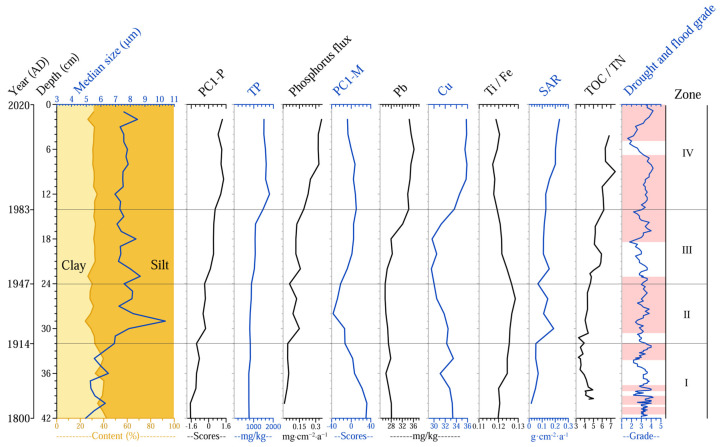
Summary of sedimentary multiproxies in Lake Huangmaotan Lake core, including median grain size/particle size distribution; historical PC1−P; total phosphorus (TP); phosphorus flux (TP/SAR); historical PC1-M; concentrations of Lead (Pb) and copper (Cu); titanium (Ti)/Iron (Fe); historical SAR; total organic carbon (TOC)/total nitrogen (TN); drought and flood grade. TOC and TN data refer to reference [39].

The first principal component axis of the plant macrofossil PCA explained 71.4% of the species variance in the plant macrofossil data, effectively indicating changes in the plant vegetation. Figure 6 shows the variations in PC1−P relative to other sedimentary indicators. The PC1−P exhibited a clear four-phase evolutionary pattern, with Zone I persistently maintaining low values, while Zones II through IV all displayed a pattern of rapid short-term increase followed by stabilization, which indicated a progressive upward trend. Heavy metal elements (Cu and Pb) displayed consistent trends. Concentrations were higher in Zone I but declined over time. In Zones II and III, concentrations gradually decreased, reaching minimum values during the 1950s–1960s, before rebounding and maintaining elevated levels in Zone IV. The PC1−P for heavy metals followed a similar decline-then-rise pattern. TP levels remained low in Zones I and II but began to increase rapidly around 1976, stabilizing at high values (1518–16,378 mg/kg) in Zone IV. The median grain size was relatively small in Zone I, increased sharply to a peak (10.4 μm) in Zone II, and then slightly declined but remained elevated overall, with the silt fraction dominating over the clay fraction. SAR showed a gradual increase throughout the sequence, with a notable rapid rise in Zone II.

### 3.6. Multivariate Correlation Analysis

**Figure 7:**
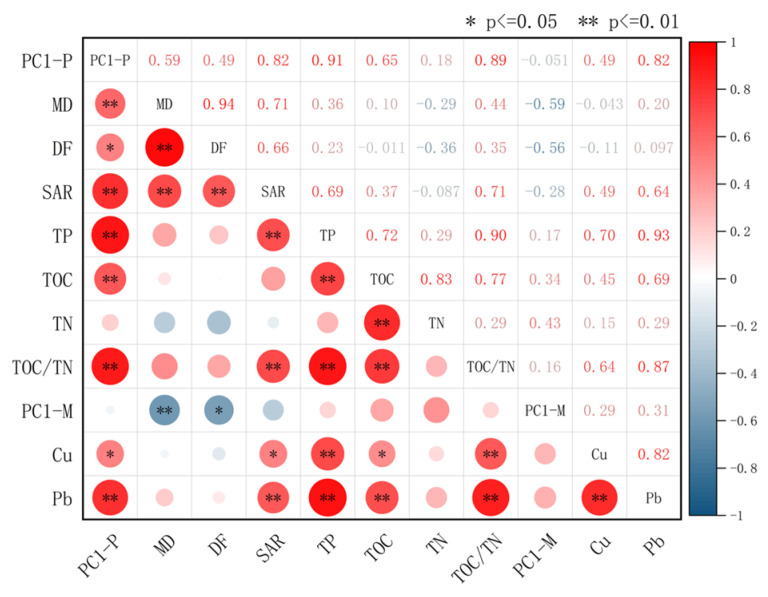
Huangmaotan Lake multi-indicator correlation heatmap. Positive (red) or negative (blue) correlations between indicators are shown by color (ranging from +1.00 to −1.00). Abbreviations: MD, median grain size; DF, drought–flood grade.

Significant correlations were observed among sedimentary indicators in Huangmaotan Lake (Figure 7), with red and blue colors representing positive and negative correlations, respectively. Deeper hues and larger shapes indicate stronger relationships. The analysis revealed statistically significant positive correlations (*p* ≤ 0.01) between drought–flood grade, median grain size, and SAR. Heavy metals Pb and Cu showed strong positive covariation (*p* ≤ 0.01), as did nutrient elements TOC and TN (*p* ≤ 0.01). Grain size exhibited a significant negative correlation with PC1−M scores (*p* ≤ 0.01) but no significant relationship with heavy metals Pb and Cu. Heavy metals (Pb, Cu), nutrients (TP, TOC, TN), drought–flood grade, grain size, and sedimentation rate all exhibited significant positive correlations with PC1−P scores. The strongest correlation was with TP (r = 0.91), followed by TOC/TN (r = 0.89).

## 4. Discussion

The aquatic macrophyte vegetation in Huangmaotan Lake has exhibited an overall shift over the past two centuries, transitioning from species preferring clear and stable water conditions toward those tolerant of hydrological disturbances and elevated nutrient levels.

### 4.1. Low Human Disturbance on Aquatic Communities During 1800–1947

Prior to 1914 (Zone I), the aquatic vegetation in the lake was dominated by *Chara* sp., a taxon characteristic for stable hydrological conditions (Figure 5), which correlated well with the time of the terminal cold phase of the Little Ice Age during the 19th century. Under this cold-phase climate regime, the watershed experienced reduced precipitation and increased aridity, leading to generally weaker hydrodynamic conditions in the lake. In lacustrine sediments, coarser grain size reflects stronger hydrodynamic forces, while finer particles indicate calmer conditions [40]. The relatively fine grain size observed in Huangmaotan Lake’s sediment core provides direct evidence of stable hydrodynamic conditions during this period (Figure 6). The benthic rosette-forming submerged macrophyte *Chara* sp. is typically adapted to oligotrophic conditions and exhibits low tolerance to both light limitation and elevated nutrient concentrations [41]. The low TP concentrations recorded in the lake sediments during this period further corroborate the oligotrophic state and clear water conditions. During this phase, the relatively stable hydrodynamic conditions prolonged the nutrient retention time, thereby enhancing the uptake and utilization of aquatic nutrients by organisms [42]. This process may have contributed to the expansion of aquatic macrophytes, resulting in luxuriant growth and relatively high species diversity of submerged vegetation during this period.

During the period 1914–1947 (Zone II), coinciding with the peak of 20th-century global warming [43], the watershed climate exhibited a distinct warm–wet trend, leading to a significant increase in flood frequency (Figure 2). Sediment records showed a marked rise in coarse particle content and median grain size, reaching peak values that indicate substantially enhanced hydrodynamic conditions during this period (Figure 6). Intense water-level fluctuations likely caused the decline and eventual disappearance of *Chara* sp., being sensitive to high water levels and increased turbulence. Meanwhile, the disturbance-adapted species *N. marina*, *H. verticillata*, and *V. natans* became the new dominant macrophytes in the lake (Figure 5). However, the exceptionally strong hydrological disturbances during this period resulted in overall low macrophyte coverage, and a similar pattern has been observed in other shallow lakes across the middle and lower Yangtze River basin during the same time period [44]. It is therefore reasonable to assume that the aquatic vegetation was primarily influenced by alterations in the lake hydrodynamics resulting from natural climate conditions, given the generally low level of human activity during this period.

### 4.2. Intensifying Anthropogenic Pressures (1947–2020)

From 1947 to 1983 (Zone III), the concentrations of the nutrients TOC, TN, and TP in the Huangmaotan sediments increased. The concentrations of the heavy metals (Pb, Cu) also increased, indicating that human activities in the watershed intensified and that more industrial and agricultural wastewater was discharged into the lake, leading to significant changes in the lake environment. Previous studies have shown that excessive heavy metals can severely impair aquatic plants by inhibiting photosynthesis, inducing oxidative stress, disrupting nutrient uptake, stunting growth, and causing genotoxicity [45]. For example, *H. verticillata*, a species that suffered drastic historic decline during this period, is highly sensitive to Cu ions. Increased Cu concentration can not only inhibit chlorophyll synthesis and cause structural damage in *H. verticillata* but also induce changes in DNA methylation of this species [46,47], which may have contributed to the reduction of *H. verticillata* in Huangmaotan Lake. The abundance of *Gloeotrichia echinulata* increased during this period (Figure 5), forming typical cyanobacterial colonies, suggesting that the lake was in the early stages of eutrophication [48,49]. As nutrient levels in the lake increased, aquatic plants obtained more nutrients. This resulted in an increase in the number and diversity of emergent plants. In 1957, a shift from natural fishing to intensive aquaculture increased both fish yields and grazing pressure on macrophytes [27]. Consequently, fish-favored *V. natans* declined, while unpalatable *N. marina* proliferated and dominated [50].

During the period 1982–2020 (Zone IV), as human activities intensified, the concentrations of heavy metals (Pb, Cu) and TP in lake sediments showed a continuous upward trend (Figure 6), reflecting rapid industrial and agricultural development in the region. Concurrently, the increasing complexity of the lake shoreline morphology (Figure 3) further demonstrated the escalating anthropogenic pressures on the lacustrine system. Since the 1980s, the Huangmaotan Lake watershed has experienced rapid expansion of aquaculture and electronics manufacturing industries [27], and this resulted in large volumes of industrial and agricultural wastewater being discharged into the Huangmaotan Lake from 1982 to 2020. With the intensified industrial and agricultural activities in the basin, the phosphorus content of sediments rapidly rose to nearly 1800 mg/kg (Figure 6), which significantly increased the nutrient load on aquatic plants. Although the oligo-mesotrophic species *N. marina* and *N. minor* remained the dominant aquatic plant, species adapted to mesotrophic to eutrophic environments began to appear in significant numbers in the lake during this stage (Figure 5). These included *V. denseserrulata* and *Potamogeton* sp. During this time, the aquatic vegetation in Huangmaotan expanded considerably.

### 4.3. Flood-Induced Macrophyte Inhibition in Huangmaotan Lake During Historical Periods

A significant positive correlation was observed between drought–flood grades and median grain size (r = 0.94, *p* ≤ 0.01), indicating that sediment grain size distribution effectively reflected flood frequency. Additionally, there was a significant positive correlation between the drought–flood grade and the PC1−P (r = 0.49, *p* ≤ 0.05), indicating that flood events had a significant impact on aquatic plant communities. Analysis of plant macrofossil data from Huangmaotan Lake (Zone I–Zone II) between 1800 and 1947 further demonstrates that aquatic plant communities in the lake were negatively impacted by flood events during this period. Specifically, before the 19th century, floods occurred less frequently, and the hydrodynamic conditions of the lake were stable. Therefore, the aquatic plant vegetation was dominated by *Chara* sp., which prefers stable hydrological conditions. However, there were several periods of frequent flooding in the 19th century (the 1810s–1820s, the 1830s–1850s, and the 1870s). During these periods, the growth of *Chara* sp. communities was inhibited, while *H. verticillata* temporarily became the dominant species in the lake (Figure 5). Extreme floods raised lake water levels and brought large amounts of sediment, severely reducing water transparency. *Chara* sp. have high light compensation points and struggle to adapt to low-light conditions, which leads to growth inhibition [51]. At the same time, the strong water disturbance caused by flooding can wash away the seed bank buried in the substrate and even cause the plant body to break [52]. However, *H. verticillata* possesses buds capable of long-term dormancy, rapid growth rates, and a photosynthetic mode similar to C4, enabling it to withstand the negative effects of flooding to some extent and recover quickly afterward [53]. However, given the frequent flooding that occurred between 1914 and 1947, the coverage of aquatic vegetation in the lake reached its lowest point in nearly two centuries. Concurrently, the population of *H. verticillata*, a species that is able to thrive in environments disturbed by flooding, experienced a substantial decline. The original dominant species *Chara* sp. underwent a gradual extinction process due to frequent extreme flooding events.

### 4.4. Inhibitory Effects of Nutrient Enrichment on Aquatic Macrophytes

Shallow lake ecosystems have two typical states, a “clear water” regime dominated by aquatic plants and a “turbid water” regime dominated by planktonic algae [54]. These regimes may shift due to external environmental factors, with nutrient enrichment being one of the most critical. Sedimentary data from Huangmaotan Lake reveal the presence of *N. marina*, *N. minor*, and *Chara* sp., recognized as indicators of a clear water regime [25,55]. These species exhibit sensitivity to nutrient inputs and flourish under oligotrophic to mesotrophic conditions, whereas eutrophic environments inhibit their growth. For instance, *N. minor*, the dominant species in Zone IV of Huangmaotan Lake, exhibits inherently low nutrient assimilation efficiency. Whole plants demonstrate deficient phosphorus translocation between roots and shoots; consequently, excessive phosphorus inputs inhibit their growth [56]. Sedimentary records from Huangmaotan Lake also reveal the presence of species adapted to meso-eutrophic conditions, such as *V. denseserrulata*, *V. natans*, and *Potamogeton* sp. Although these plants exhibit high nutrient tolerance, they decline under conditions of excessive nutrient enrichment. This is because enhanced nitrogen and phosphorus levels stimulate epiphytic and planktonic algal growth, reducing water clarity and light availability for submerged vegetation, ultimately leading to their reduction or local extinction and potentially triggering a transition to a turbid water regime [57]. In the past five decades, the majority of lakes located within the middle and lower reaches of the Yangtze River have been impacted by the escalation of anthropogenic activities. The influx of excess sewage has been a primary factor in the eutrophication of these water bodies, leading to a substantial decline in aquatic vegetation across a considerable expanse [58,59]. For example, since the 1980s, Tai Bai Lake, a typical shallow lake in the middle and lower reaches of the Yangtze River, has been accompanied by the rapid development of industry and agriculture, and the degree of nutrient enrichment in the lake has been increasing (Sediment-P about 1200 mg/kg), which ultimately led to the decline of aquatic vegetation in large areas, and the lake ecosystem was transformed into a turbid water regime [60]. Huangmaotan Lake also experienced a significant increase in nutrients entering the lake during this period (Sediment-P about 1500 mg/kg), but so far the aquatic plant vegetation in this lake is still gradually expanding and does not show a trend of decline.

### 4.5. Combined Effects of Flood Disturbances and Nutrient Enrichment Prompt Aquatic Vegetation Expansion

Flood disturbance and nutrient enrichment have been shown to negatively affect the growth of aquatic plants in lakes [15,59]. However, analysis of plant macrofossil data from Huangmaotan Lake indicates that the combined effect of these two factors can sometimes stimulate growth, contradicting the conventional wisdom that two negatives make an affirmative.

The sedimentary record of Huangmaotan lake indicates that nutrient inputs mitigated the negative effects of flood disturbance on aquatic plants. Drought and flood class calculations show that the 1970s–2000s was the period with the most consecutive flood events in the Huangmaotan Lake watershed over the last 200 years (Figure 2). Remote sensing data also reveals a significant expansion of the lake area from the 1970s to the 1980s (Figure 3). Despite more intense and frequent flood disturbances from 1983–2020, the aquatic plants in Huangmaotan Lake experienced a period of growth and prosperity, the most significant in the last 200 years. Wheeler (1999) has suggested that increased nutrient inputs may help offset the negative effects of flooding on the growth of fast-growing plants [61]. Under flooded conditions, aquatic plants typically adapt by enhancing photosynthetic efficiency through stem elongation and increased allocation to aboveground biomass to capture more light [62,63]. However, flooding often suppresses root activity by reducing oxygen diffusion, impairing nutrient uptake and thus limiting sustained aboveground growth [64]. Furthermore, elevated hydrodynamic forces may damage root tissues, causing aquatic plants to prioritize nutrient allocation to roots to maintain sustained nutrient acquisition for the whole plant. This reallocation, nevertheless, results in an overall reduction in plant biomass [65]. Conversely, a high-nutrient environment can effectively mitigate these limitations. Sufficient nitrogen and phosphorus inputs provide essential material for the morphological adaptation (e.g., proliferation of aboveground structures) of aquatic plants. This increase in aboveground structures enhances their light capture capacity, thereby increasing overall biomass [66,67]. Additionally, nutrient enrichment accelerates vascular tissue development (e.g., promoting sclerenchyma differentiation), significantly improving stem rigidity and mechanical strength [68]. This enhancement consequently bolsters plant resistance to breakage under hydrodynamic stress.

The species *V. denseserrulata* and *P. australis*, which expanded rapidly in Huangmaotan Lake during 1983–2020, have been experimentally studied to confirm that their growth is indeed promoted by combined effects of flooding and nutrient enrichment. *Vallisneria* sp., a common perennial clonal macrophyte, adapts to extreme environments through strategic placement of ramets into favorable resource patches, a process known as clonal foraging behavior, which requires substantial nutrient investment to sustain [69]. Under the previous low nutrient input conditions at Huangmaotan Lake, the growth of *V. denseserrulata* was restricted, and its plant debris only occasionally appeared in the sediment column. However, during periods of increased nutrient input in Huangmaotan Lake, *V. denseserrulata* could utilize the abundant nutrients and begin clonal reproduction. This effectively mitigated the negative impacts of floods and even stimulated their expansion under flood conditions. This phenomenon was also demonstrated in simulation experiments conducted by Zhi et al. [70]. *P. australis*, an expanding ecotonal species inhabiting lake–wetland interfaces, exhibits high sensitivity to flooding impacts. Its rhizomes cannot survive under the anoxic conditions induced by floods, ultimately leading to plant mortality [71,72]. However, with supplemental nutrient inputs following root uptake of nitrogen by *P. australis*, the absorbed nitrogen induces formation of adventitious roots and aerenchyma, thereby improving oxygen transport efficiency in hypoxic environments [73]. Studies by Tang et al. demonstrate that moderate nitrogen influx during flooding promotes root biomass accumulation and increases the below/aboveground biomass ratio. Through resource reallocation prioritizing root investment, this strategy enhances anchorage capacity and nutrient acquisition efficiency, ultimately facilitating early-stage establishment of *P. australis* [74].

During the period from 1983 to 2020 (Zone IV), Huangmaotan Lake exhibited a rapid increase in nutrient inputs. However, contrary to expectations, the aquatic macrophyte vegetation flourished significantly, with the oligo-mesotrophic indicator species *N. minor* gradually becoming dominant (Figure 5). The predominance of *N. minor* suggests that the lake’s overall trophic status remained within the oligo-mesotrophic range during this phase, maintaining relatively clear water conditions. This paradoxical phenomenon (i.e., the apparent discrepancy between elevated nutrient levels in sediment and relatively low nutrient concentrations in water column) may be closely linked to long-term flood disturbances in Huangmaotan Lake. During flood events, the Yangtze River water massively intrudes into Huangmaotan Lake. Given the lake’s limited water storage capacity, a single flood event can replace the majority of its water volume, thereby maintaining relatively low nutrient concentrations in the water column. Simultaneously, suspended particulate matter (especially the fine-grained fraction) introduced by floodwaters facilitates the deposition of dissolved nutrients into sediments through adsorption–sedimentation processes, resulting in a relative decrease in water column nutrient concentrations and a corresponding increase in sediment nutrient levels [75]. For example, research by Ji et al. demonstrated that during the wet season, detrital suspended particulate matter carried by floods from the Miju River acted as an effective phosphorus sink. It adsorbed up to 45% of dissolved inorganic phosphorus, thereby reducing dissolved inorganic phosphorus loading into Lake Erhai [76]. Under this oligotrophic water column but nutrient-enriched sediment regime, competitive growth of phytoplankton is suppressed, while the sediment provides substantial substrate nutrient support for root uptake by submerged macrophytes, collectively creating an ecological niche conducive to aquatic vegetation expansion [77].

### 4.6. Dam Effects

Since 1950, over 50,000 dams have been constructed across the middle and lower Yangtze River basin, profoundly altering the ecohydrological regimes of downstream lakes [78]. Studies on Yangtze River floodplain lakes show that stabilized hydrological regimes resulting from dam and sluice construction have created favorable conditions for aquatic macrophyte expansion. This has driven lacustrine vegetation communities into a historical proliferation period with progressive succession toward canopy-forming species (e.g., *Myriophyllum spicatum* and *Ceratophyllum demersum*) [23]. However, this overly stable hydrological environment accelerated nutrient enrichment, ultimately leading to a significant decline in lake aquatic plant communities in recent decades [25,79]. The Huangmao Dam was built in Huangmaotan Lake in 1934, and the dam raising and reinforcement project was completed in 1956 [27], effectively reducing the hydrodynamic pressure of the lake. Nevertheless, sediment median grain size in Huangmaotan Lake remained stable between 7 and 8.6 μm after the 1950s (Figure 6). In comparison, Liangzi Lake, another Yangtze floodplain lake, maintained sediment median grain sizes below 4 μm throughout the past century [25], indicating that the construction of the Huangmao Dam did not significantly reduce hydrodynamic conditions in Huangmaotan Lake, a phenomenon closely related to the hydrological characteristics of small lakes [80]. Consequently, the aquatic macrophyte vegetation in Huangmaotan Lake remained dominated by disturbance-adapted species (*N. marina* and *N. minor*) after the 1950s, without developing a canopy-forming submerged vegetation assemblage. This unique hydrological regime created by the Huangmao Dam provided balanced hydrodynamic conditions that enabled synergistic interactions between flood disturbances and nutrient enrichment, preventing extreme dominance by single environmental factors while collectively promoting macrophyte expansion.

### 4.7. Implications for Lake Management

Traditionally, the Chinese government implemented a “pollution source control and interception” strategy to address lake eutrophication, yet substantial financial investments failed to yield effective remediation outcomes [81]. In recent years, ecological restoration and long-term governance strategies have gained prominence, with the rehabilitation of lacustrine macrophyte communities and ecosystem restructuring emerging as one of the most effective lake remediation approaches [82]. However, failure to properly understand whether aquatic macrophytes historically occurred in the lake and how they respond to environmental conditions may ultimately lead to ineffective restoration outcomes or even unintended biological invasions. This study provides comprehensive and continuous records of vegetation community changes in Huangmaotan Lake, demonstrating that plant macrofossil preserved in sediments can scientifically reconstruct the historical evolution of aquatic macrophyte communities. These findings offer fundamental scientific references for understanding lake ecosystem dynamics and establishing targeted restoration objectives and pathways for aquatic vegetation rehabilitation.

Notably, the plant macrofossil record from Huangmaotan Lake shows that under high-nutrient conditions, maintained seasonal flood disturbances can not only counteract the negative impacts of nutrient enrichment on aquatic macrophytes but also promote their expansion, sustaining a relatively clear water ecosystem. Drawing on sedimentary evidence from Huangmaotan Lake, effective lake restoration in highly eutrophic systems subjected to intense anthropogenic pressures should prioritize enhancing river–lake connectivity and reestablishing seasonal flood disturbances while concurrently minimizing external inputs of nutrients and heavy metals. For lake systems experiencing excessive flood disturbances, flood impacts should be managed through regulation rather than by severing river–lake linkages. This nature-based approach leverages the combined effects of flood pulses and nutrient dynamics to more effectively drive macrophyte recovery and ecosystem rehabilitation. These findings provide critical insights for the ecological restoration of Yangtze River floodplain lakes and lentic systems facing similar challenges worldwide.

## 5. Conclusions

We reconstructed the successional dynamics of aquatic macrophyte communitiy over the past two centuries in Huangmaotan Lake, a Yangtze River floodplain lake, through integrated analysis of plant macrofossils and physicochemical indicators preserved in sedimentary records. The aquatic macrophyte community in Huangmaotan Lake has transitioned from species-adapted to stable, oligotrophic conditions to those favoring disturbed, oligo-mesotrophic environments. This successional shift was jointly driven by hydrological changes and increased nutrient inputs. Analysis of historical data combined with the sedimentary record, at a centennial scale, reveals how flood disturbances and nutrient enrichment synergistically promoted macrophyte expansion while maintaining clear water conditions. These findings provide valuable insights for ecological restoration and eutrophication management in Yangtze River floodplain lakes and similar freshwater systems worldwide.

## Figures and Tables

**Table 1:** Historical changes in lake area and shoreline of Huangmaotan Lake.

Date	Lake Area (km^2^)	Shorelines (km)
November 2020	7.485	25.414
November 2010	7.551	31.979
November 2000	6.977	25.271
December 1985	6.590	19.209
December 1973	4.812	13.749

## Data Availability

The data supporting the findings of this study are available from the corresponding author upon reasonable request.
